# The role of BRCA1-IRIS in ovarian cancer formation, drug resistance and progression

**DOI:** 10.18632/oncoscience.308

**Published:** 2016-06-06

**Authors:** Wael M. ElShamy

**Affiliations:** Cancer Institute, University of Mississippi Medical Center, Jackson, MS 39216, USA

**Keywords:** BRCA1-IRIS, ovarian cancer, anoikis, therapy, metastasis

## THE OVARIAN CANCER ONCOGENE BRCA1-IRIS

**Figure 1:**
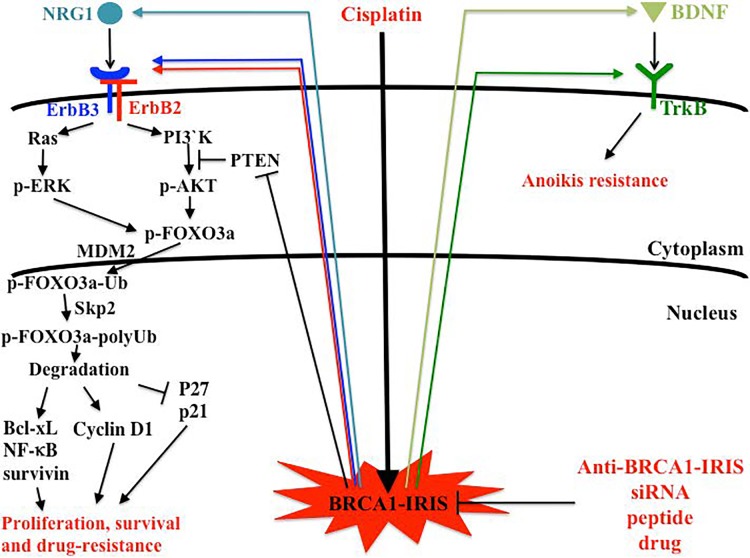
Potential impact of strategies that target BRCA1-IRIS on the treatment of ovarian cancer BRCA1-IRIS overexpression impacts on several proliferation, survival, anoikis and drug resistance pathways. Conventional therapies, such as cisplatin, while kill some of tumor cells, two major obstacles are limiting success in BRCA1-IRIS overexpressing tumors. First, the ability of BRCA1-IRIS to promote drug resistance, so many overexpressing cells will survive these cytotoxic treatment, and second is the potential of cisplatin, especially at low concentration to elevate BRCA1-IRIS expression in no/low BRCA1-IRIS expressing cells, thus generating a whole new cancer cells with drug resistance and aggressiveness potential. The use of BRCA1-IRIS specific inhibitor(s) modalities would reduce these cells resistance to therapy and their ability to relapse, spread, disseminate and colonize secondary sites.

The BRCA1-IRIS protein was identified in 2004 [1] and later found to be frequently upregulated in multiple sporadic tumor types, including breast and ovarian cancers [2, 3]. In ovarian cancer samples, BRCA1-IRIS expression increases with tumor progression together with survivin [3]. This trend was positively correlated with significant decrease in the level of total FOXO1 and FOXO3a and increase in the phosphorylated form of these proteins and their cytoplasmic sequestration (Figure 1). BRCA1-IRIS overexpression also impacted negatively on the expression of PTEN, which is a potent inhibitor of AKT [3]. As a consequence, loss of PTEN function led to activation of the phosphoinositide 3-kinase (PI3K)-AKT1/2 pathway in BRCA1-IRIS overexpressing cells, which stimulated their growth, survival and drug (e.g., cisplatin) resistance (Figure 1). These data of BRCA1-IRIS and associated oncogenic pathways, has caused great interest in elevated BRCA1-IRIS expression as a biomarker for ovarian cancer early lesions.

## BRCA1-IRIS AS AN INDUCER OF CISPLATIN RESISTANCE

As mentioned above the upregulation of BRCA1- IRIS promotes PTEN downregulation leading to AKT activation, FOXO1 and FOXO3a activation and survivin upregulation (Figure 1). These cells are intrinsically resistance to physiological concentrations of cisplatin, raising the prospective of treatment failure for ∼50% of all ovarian cancer patients overexpressing BRCA1-IRIS (Figure 1) [3]. Moreover, low concentration cisplatin upregulates BRCA1-IRIS expression in low expressing cells. This disturbing trend suggest that chemotherapy, e.g., cisplatin generates BRCA1-IRIS overexpressing cells in the other 50% of patients with tumor expressing low level of the protein. Alternatively, in residual tumor cells after surgery leading to drug resistance recurrences (Figure 1) [3]. This could explain the high percentage of the rapid development of the drug resistance recurrences in the majority of patients (larger number than the 50% we reported initially overexpressing BRCA1-IRIS), which eventually kill many patients (Figure 1) [3].

## BRCA1-IRIS AS A DRUG TARGET FOR OVARIAN CANCER

The discovery of BRCA1-IRIS [1] was accompanied by detection that deleting the intron 11 domain of BRCA1- IRIS abrogated the oncogenic functions of BRCA1-IRIS, at least *in vitro* [3]. Based on these data and the fact that this domain both very short and does not resemble any know domain structure in the protein database, we proposed that BRCA1-IRIS does not act as an oncogene by itself. We proposed that the protein uses this domain to establish interactions with protein(s) and the complex(s) act as an oncogene. This was confirmed by the fact that a peptide resembling the intron 11 domain inhibited growth of ovarian cancer cells *in vitro* and *in vivo*. In addition, peptide treated cells were much more sensitive to death by cisplatin even at low concentration, *in vitro* and *in vivo*. The peptide enhanced BRCA1-IRIS degradation in cancer cells, suggesting that free BRCA1-IRIS is unstable. This data opens up the possibility that this peptide could be used therapeutically. In this regards, while ovarian cancer cells generated tumors in SCID mice, and cisplatin was unable to significantly affect the growth of these tumors, tumors in peptide treated mice significantly regressed. The effect of this peptide was even seen in combination treatment with cisplatin, suggesting an *in vivo* sensitization of ovarian cancer cells to cisplatin by inhibiting BRCA1- IRIS survival pathways (Figure 1) [3]. In keeping with that in this *in vivo* model, unlike cisplatin treated tumors that should increase BRCA1-IRIS and survivin expression, treatment with BRCA1-IRIS peptide alone or in combination with cisplatin significantly abrogated BRCA1-IRIS and survivin expression in the regressed tumors [3].

## BRCA1-IRIS AS A PROGNOSTIC BIOMARKER

High BRCA1-IRIS expression is associated with response to conventional standard of care chemotherapy (manuscripts in preparation). Patients with BRCA1-IRIS overexpressing ovarian tumors have also been shown to have shorter survival compared with cases with low BRCA1-IRIS expressing tumors [3]. It is perhaps possible to suggest that as a prognostic for outcome following therapy in ovarian cancer, BRCA1-IRIS expression could be measured. BRCA1-IRIS overexpression also shows promise as a predictive marker for targeted therapeutic agents including anti-ERBB2, ERBB3 and TrkB [3–6]. The utility of using BRCA1-IRIS as a biomarker of diagnosis and prognosis or predictor for drug response clearly needs further investigation. Only through a greater understanding of the function played by BRCA1-IRIS in regulating various biological functions will its role as a biomarker and treatment target be fully realized. Additionally it will be imperative to evaluate the mechanism cellular and genomic insults upregulate BRCA1-IRIS with, which could also be targeted to increase the ovarian cancer treatments efficacies in the clinic.
